# A microarray analysis of gene expression in the free-living stages of the parasitic nematode *Strongyloides ratti*

**DOI:** 10.1186/1471-2164-7-157

**Published:** 2006-06-19

**Authors:** Fiona J Thompson, Gary LA Barker, Louise Hughes, Clare P Wilkes, Jane Coghill, Mark E Viney

**Affiliations:** 1 School of Biological Sciences, University of Bristol, Woodland Road, Bristol, BS8 1UG, UK

## Abstract

**Background:**

The nematode *Strongyloides ratti *has two adult phases in its lifecycle: one obligate, female and parasitic and one facultative, dioecious and free-living. The molecular control of the development of this free-living generation remains to be elucidated.

**Results:**

We have constructed an *S. ratti *cDNA microarray and used it to interrogate changes in gene expression during the free-living phase of the *S. ratti *life-cycle. We have found very extensive differences in gene expression between first-stage larvae (L1) passed in faeces and infective L3s preparing to infect hosts. In L1 stages there was comparatively greater expression of genes involved in growth. We have also compared gene expression in L2 stages destined to develop directly into infective L3s with those destined to develop indirectly into free-living adults. This revealed relatively small differences in gene expression. We find little evidence for the conservation of transcription profiles between *S. ratti *and *S. stercoralis *or *C. elegans*.

**Conclusion:**

This is the first multi-gene study of gene expression in *S. ratti*. This has shown that robust data can be generated, with consistent measures of expression within computationally determined clusters and contigs. We find inconsistencies between EST representation data and microarray hybridization data in the identification of genes with stage-specific expression and highly expressed genes. Many of the genes whose expression is significantly different between L1 and iL3s stages are unknown beyond alignments to predicted genes. This highlights the forthcoming challenge in actually determining the role of these genes in the life of *S. ratti*.

## Background

Parasitic nematodes have complex life-cycles that are affected and controlled by factors both within and outwith their hosts. In the genus *Strongyloides*, the life-cycle, unusually, includes both an obligate female-only parasitic generation and a facultative dioecious adult free-living generation. In recent years there has been an increasingly detailed understanding of the factors that affect the development of the free-living phase of this life-cycle, particularly for the parasites of rats, *S. ratti *[1].

**Figure 1:**
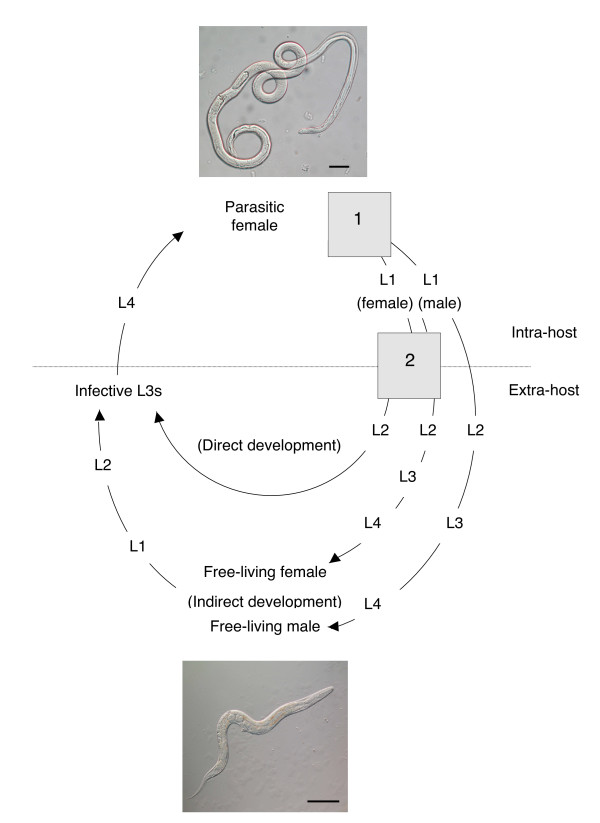
The life-cycle of *S. ratti *with two discrete developmental switches, shown as grey boxes: (1) a sex determination event and (2) a female-only developmental switch. L, denotes larval stages, as numbered. An adult parasitic female (top) and free-living adult female (bottom) are shown; bars = 100 μM. After [4].

*S. ratti *parasitic females lie embedded in the mucosa of the small intestine of their host. These females reproduce by mitotic parthenogenesis and lay eggs that pass with the host faeces into the environment, where the development of the free-living stages occurs [2]. Direct development occurs when genetically female first-stage larvae (L1) moult twice to develop into infective third stage larvae (iL3s) which can infect new hosts by skin penetration. In indirect development, both genetically male and female L1s moult four times into adult free-living males and females, respectively. These free-living adults undergo sexual reproduction, the female lays eggs that then (as for direct development, above) develop into iL3s (Figure 1) [3].

The control of the developmental fates of the larvae of the free-living generation occurs by the action of a male/female genetic sex determination event [4] and a subsequent female-only developmental choice. In this female-only choice, genetically female larvae can develop directly into iL3s or develop indirectly into free-living adult females. Analysis of the proportion of larvae that develop by these two developmental routes shows that as an *S. ratti *infection progresses two changes occur. Firstly, the proportion of larvae that develop into free-living males increases, *i.e. *the sex ratio becomes more male biased. Secondly, that the proportion of female larvae that develop indirectly into free-living females is favoured, *i.e. *the female-only developmental choice becomes more biased towards the development of adult free-living females [5]. That these two changes occur as an infection progresses in immunologically normal rats, but that these do not occur in immunodeficient rats, suggests that this is controlled by the host's anti-*Strongyloides *immune response. The temperature, external to the host, at which larvae develop also affects the female-only developmental choice, with indirect development of female larvae favoured at greater temperatures. The effects of the host immune response and of temperature external to the host interact, such that the temperature sensitivity of developing female larvae is greater in larvae passed from hosts that have an anti-*S. ratti *immune response [5]. In addition to these significant effects of within- and outwith-host environmental conditions and their interaction, there is a strong *S. ratti *genetic component. This genetic effect is seen both as different developmental propensities of different isofemale lines and as the response of isofemale lines to selection for different developments [6,7]. The genus *Parastrongyloides *is the closest relative to *Strongyloides *[8,9]. In the most thoroughly investigated species, *P. trichosuri*, there is a facultative dioecious free-living adult generation, similar to that of *Strongyloides*; however, the parasitic generation is also dioecious, unlike *Strongyloides *[10,11].

For many species of parasitic nematodes, including *Strongyloides*, the infective stage is a third larval stage (L3). Many analogies have been drawn between iL3s of parasitic nematodes and the dauer larvae of free-living nematodes such as *C. elegans *[12,13]. Dauer larvae of *C. elegans *are long-lived arrested stages formed under conditions of high con-specific population density and low food availability [13]. The particular similarities of iL3s of parasitic nematodes and of dauer larvae of *C. elegans *and other free-living nematodes are that they are both third larval stages, they are arrested in their development, and they are non-feeding, environmentally resistant stages with specialised morphology. Both iL3s and dauer larvae persist in the environment until specific cues signal the resumption of development; for parasitic nematodes this is the infection of a new host, for *C. elegans *this is the availability of food. The transcription rates in *C. elegans *dauer larvae are approximately six-to-seven fold lower compared with actively growing non-dauer stages [14]. More recently, SAGE and microarray analysis have identified the expression of dauer-specific or -enriched genes, including genes that appear to only be transcribed upon exit from the dauer stage. This suggests that dauer larvae are more transcriptionally active than hitherto thought [15,16].

The existence of dauer larvae suggests the possibility that they are at least part of the means by which the parasitic nematode lifestyle evolved from a free-living one. For *C. elegans*, the genetic and molecular genetic control of the commitment to develop as dauer larvae is well characterised and includes a TGF-β-like pathway, an insulin-like pathway and a guanyl cyclase pathway [13]. Comparisons of *S. stercoralis *(a parasite of humans) L1- and iL3-specific or -biased genes with *C. elegans *dauer or non-dauer-specific or -biased genes showed greater significant alignment of the *S. stercoralis *L1 and *C. elegans *non-dauer genes (but not other combinations) than expected by chance [17]. These results suggest that there is some transcriptional conservation of genes involved in early larval growth, but that there is no such conservation between dauer larvae of *C. elegans *and iL3s of *S. stercoralis*; this, therefore, does not obviously support the hypothesised evolutionary link between iL3s of parasitic nematodes and dauer larvae of free-living nematodes. For some parasitic nematodes, genes putatively homologous to the TGF-β-like genes involved in the initiation of *C. elegans *dauer larva development have been identified and their life-cycle expression profiles determined [18-22]. For four species of parasitic nematodes, this has shown that these apparently homologous genes have a similar (or at least analogous) expression pattern through their respective life-cycles, but one that is different to that of *C. elegans*. These results may, therefore, suggest that this gene has evolved a parasitism-specific role or function [23]. This is an example of the particular challenge of beginning to understand how the role and function of genes has both been maintained and changed during the evolution of parasitism [8,24].

In recent years there have been extensive EST-based gene discovery programmes for parasitic nematodes. To date, some 21 genera of parasitic nematodes, including *S. ratti *and *S. stercoralis*, have been analysed which has generated *c*. 300,000 ESTs [17,25-27]. An analysis of these data from 30 species of nematodes (28 of which are parasitic) has shown that there are significant differences between different nematode species and groups. For example, more than half the predicted genes from nematodes appear to be unique to the phylum and 23% of the predicted genes are unique to the species from which they were found [25].

The enormous quantity of data available for parasitic nematodes now needs to be used to understand the biology of these important pathogens and to use such understanding and the genomic data directly, to identify chemotherapeutic and vaccination targets. This genomic-scale work is now beginning to be able to complement the extensive genetic, genomic and post-genomic analyses of the model free-living nematodes *C. elegans *and *C. briggsae *[28,29]. For these species, there is now a very good understanding of many aspects of its development and life, which in contrast to the situation with many, if not most, parasitic nematodes.

In the EST analysis of *S. ratti*, 14,761 ESTs (*i.e. *70% successful, high quality sequence data from *c*. 20,000 reads) were obtained from five cDNA libraries encompassing the free-living (L1, L2 and mixed iL3/free-living adult) and parasitic female (6 and 15 days p.i.) stages of the life-cycle (Figure 1). Bioinformatic analyses reduced these ESTs into 5,237 contig sequences where each contig contains multiple ESTs that represent apparently identical and, or overlapping transcripts. These contigs were further grouped into 4,152 clusters, which are likely to contain splice-variants, highly similar gene-family members, alleles and polymorphisms. Assuming that *S. ratti *has approximately 20,000 genes (as does *C*. *elegans*) [28], then these clusters represent approximately 20% of *S. ratti'*s genes. Comparison of these clusters to all available sequences showed that 25% (*i.e. *1,065) of clusters had no significant alignments and therefore may represent genes newly discovered in *S. ratti *and, or *S. ratti*-specific genes, or 5' and 3' untranslated regions that are known to show very little homology between species [27,30]; the remaining 75% had significant alignments to proteins from *C. elegans*, other nematodes or non-nematodes. Analysis of the representation of the ESTs, by their cluster, in the different cDNA libraries was used as a measure of gene expression. Of the 4,152 clusters, 1,413 contain more than one EST; analysis of the distribution of the ESTs of these clusters between the four different life-cycle stages available, showed that 44% of clusters (*i.e. *623) are expressed in more than one life-cycle stage. Analysis of genes expressed at high levels (as measured as the number of ESTs within any one cluster) and a comparison of the occurrence of these between the free-living and parasitic stages shows that 30 clusters account for 38% of all expression and that 26 of these clusters have significantly different levels of expression between the free-living and parasitic stages of the life-cycle [27].

Studies of gene expression during the life cycle of several parasitic nematodes have recently been undertaken. For *Brugia malayi*, an oligonucleotide array representing 3,569 clusters generated from ESTs sequenced from 15 cDNA libraries that represent the major *B. malayi *life cycle stages [31] was probed using cDNA from adult males and females. This study identified 1,170 clusters that showed sex-dependent expression. Similarly, for *Trichostrongylus vitrinus*, 1,377 genes with sex-dependent expression were identified by microarray analysis [32]. For *Ascaris suum*, a DNA array of 1,920 ESTs was analysed to identify genes that had co-ordinated changes in expression during the within-host L4 stage [33]. In contrast to this work with parasitic nematodes, extensive microarray and SAGE analysis has been carried out with *C. elegans*, particularly comparing gene expression in different life-cycle stages [15,34,35].

Here we report the construction of a DNA microarray of available *S. ratti *ESTs, which we have used to begin to explore changes in gene expression in different aspects of the development of the free-living stages of the *S. ratti *life-cycle (Figure 1). The broader rationale of this work was to begin to investigate and understand the role of these recently discovered genes in the life of *S. ratti*, which for the vast majority are, so far, totally unexplored. In this work we have, specifically, compared gene expression between (i) free-living L1s and iL3s and (ii) free-living L2s destined to develop directly into iL3s and L2s destined to develop indirectly into free-living adults (Figure 1) [5,7,27]. *A priori*, the L1 v iL3 comparison is seeking substantial differences between morphologically and biologically distinct life-cycle stages; the L2 direct v L2 indirect comparison is seeking small differences, *i.e. *between larvae with different future development. For this latter comparison, we hypothesised that by discovering genes whose expression was significantly altered at the beginning of these different routes of development, that this may identify key processes and pathways that control direct and indirect development of *S. ratti*. For the comparison of gene expression in L1 and iL3 stages, we hypothesised that this would identify genes central to larval growth (*i.e. *genes with greater expression in L1s) and genes involved in the induction and maintenance of the arrested state of iL3s (*i.e. *genes with greater expression in iL3s). This latter group of genes also allows a comparison of the transcriptome of *C. elegans *dauer larvae and of *S. ratti *iL3s. Furthermore, the *S. ratti *L1 v iL3 comparison of gene expression is also analogous to the EST representation-based analyses of gene expression of these stages in *S. stercoralis *[17].Overall, these studies also allowed an extensive series of experiments, validating this microarray approach with *S. ratti *and exploring the genetic basis of the development of the free-living generation.

## Results and discussion

### L1 v iL3

Gene expression was compared in L1 passed in faeces and in iL3s (Figure 1). The microarray used consisted of some 9,534 individual clones which are represented by 2,742 contigs and 2,590 clusters. The resulting microarray expression data are analysed at these three levels to quality control the data generated and to investigate and compare gene expression in these different life-cycles stages.

### L1 v iL3: data quality assurance

26 microarray slides (*i.e. *13 dye swap experiments) were probed using four different biological replicates, 21 of these slides passed our quality controls and went forward for analysis. Across all the biological and technical replicates data were available for 7,446 of the 9,984 arrayed features. 1,903 ESTs were identified whose expression was significantly different between the L1 and iL3 stages (p ≤ 0.05). This number is greater than the 372 significant results that would be expected by chance (7,446 × 0.05). Of these 1,903, the expression of 1,244 was significantly greater in the iL3 stage and 659 significantly greater in the L1 stage, of which 147 and 217 ESTs were expressed two or more-fold greater in the iL3 and L1 stages, respectively (Table 1). The hybridization of control 'spikes' 3- and 10-fold different between samples resulted in mean observed differences in expression of 4.8 and 7.7, respectively. These measurements suggest that the dynamic range over which the observed experimental data fall is broadly correct though, somewhat constrained compared with the actual differences in observed expression.

We wished to assess the reproducibility of expression data between individual arrayed ESTs within their respective contigs and clusters. To do this, for each of these 1,903 ESTs that were significantly differentially expressed, we also analysed the expression data for other contig and cluster members (excluding single-EST clusters) present on this microarray to which these 1,903 ESTs belonged. The 1,903 ESTs belonged to 988 contigs and 936 clusters; 403 ESTs were single-EST contigs and clusters. For the 1,500 (*i.e. *1,903 - 403) non-single EST contigs and clusters we determined the proportion of ESTs belonging to each contig and cluster which (i) produced hybridization data that passed the quality control threshold and (ii) the proportion of these that were also significantly different between the L1 and iL3 stages. These measures are an assessment of the reproducibility of the expression ratio data between contig and cluster members. A mean of 99.2% and 99.4% of ESTs of these contigs and clusters, respectively, produced hybridization data that passed the quality control threshold; a mean of 55.0% and 54.8% of the ESTs of these contigs and clusters, respectively, were significantly (p ≤ 0.05) differently expressed between the L1 and iL3 stages. This generally high level of concordance of the measured expression of individual ESTs suggests that data arising from individual arrayed ESTs can be used as criteria for selecting contigs and clusters for further analysis. Similarly, the generally high level of congruence of detection of significantly different expression, also suggests that robust measurement of differential gene expression is possible with these microarrays. Individual arrayed EST members of individual contigs and clusters are diverse with respect to the length of sequence arrayed which is likely to contribute substantially to inter-EST variation in observed expression.

To further investigate the relationship of measures of expression for contigs and their respective clusters, we identified contigs whose ratio of expression (formally, the greatest mean fold difference of component ESTs that were significantly differently expressed between the L1 and iL3 samples) was greater than or equal to two. This resulted in 50 contigs whose expression was 'up' in this manner in the L1 stage and 69 contigs 'up' in the iL3 stage (Table 1). These 119 contigs represent 99 clusters, 18 of which (for both the L1-up and the iL3-up contigs) are multi-contig clusters. We interrogated the microarray expression data for the other contigs of these multi-contig clusters. This analysis showed that within 8 clusters, more than one component contig produced microarray hybridization data above the quality threshold (above), 10 did not. Overall, these 8 multi-contig clusters consisted of a total of 46 contigs, of which the ratio of the expression between the L1 and iL3 stages of 28 contigs (61%) was directionally concordant within a cluster; only 4% were directionally discordant. The remaining 35% of contigs showed directionally concordant expression, though the difference between the L1 and iL3 stages was not statistically significantly different. This further suggests both that the gene expression measured by these microarrays is a robust measurement of actual gene expression and that both the computationally created contigs and clusters [27] are accurate representations of actual units of expression [see Additional files 1 and 2].

Our previous analysis of the *S. ratti *ESTs considered large clusters, *i.e. *clusters containing the greatest number of ESTs across all *S. ratti *life-cycle stages [27]. Analysis of the representation of ESTs between the different cDNA libraries suggested that 28 of the 30 biggest clusters were expressed in free-living stages [27]. These 28 clusters are represented by 1,922 ESTs, which is 26% of the total 7,490 ESTs present on this microarray. We were concerned that highly represented contigs and clusters may be identified as significantly different between the L1 and iL3 stages through purely stochastic events relating to the size of these contigs and clusters. We investigated this by analysis of all ESTs belonging to these top 30 clusters [27]. This showed that for the 28 largest clusters on this microarray a mean of 99.8% of the ESTs from these clusters, that were present on the microarray, produced hybridization data above the quality threshold, of which 26.8% were significantly different between the L1 and iL3 stages. For all of the remaining clusters (*i.e. *other than the largest clusters) for which there were data across all the biological and technical replicates (*i.e. *5,568 ESTs), a mean of 99.3% of the ESTs of each cluster produced hybridization data above the quality threshold of which 24.9% were significantly different between the L1 and iL3 stage. This high degree of similarity between the hybridization behaviour of ESTs of large clusters and all the EST data, shows that ESTs of large clusters do not behave any differently. These results therefore suggest that high representation of a cluster does not, of itself, result in the generation of significant results from this microarray. Of these 30 largest *S. ratti *clusters [27], the microarray expression data showed that four were significantly differently expressed between L1 and iL3 stages with a two-fold or greater difference: L1-up SR00012 2.27-fold, SR01068 2.08-fold, SR00026 2.04-fold; iL3-up SR00369 3.60-fold.

Previously, putative stage (L1 or iL3) specific gene-expression had been determined based on the representation of ESTs among cDNA libraries [27]. We wished to determine whether this was supported, or not, by the microarray data. To do this we investigated the concordance of the L1 v iL3 microarray results with the occurrence of ESTs in the L1 or mixed iL3/free-living adult library [27]. Specifically, from the microarray results, for the 119 contigs significantly differently expressed between the L1 and iL3 stages (with a two or more-fold difference in expression) we determined from the observed cDNA library representation, the proportion of contigs for which this was (i) concordant (*i.e. *for those contigs identified by these microarray results as being L1-up, concordance was judged to have occurred when the contig in question was represented in the L1 library, but absent from the mixed iL3/free-living adult library or present with a lower frequency, and *vice versa *for the iL3-up contigs); (ii) discordant (*i.e. *for those contigs identified by these microarray results as being L1-up, discordance was judged to have occurred when the contig in question was represented in the mixed iL3/free-living adult library, but absent from the L1 library or present with a lower frequency, and *vice versa *for the iL3-up contigs) or (iii) equal. Combining the results for the L1- and iL3-up contigs showed that there was 39.5% discordance, 29.5% concordance and 31% equality. This high level of discordance suggests that the use of representation of ESTs in different cDNA libraries may not be a good measure of differences in expression between different life-cycle stages or between different samples.

We used reverse transcriptase-PCR (RT-PCR) to confirm the microarray expression data for a selection of genes with a range of L1 : iL3 differences in expression. The mean RT-PCR expression ratios of the 38 L1- or iL3-up contigs together with their microarray mean cluster and contig expression ratios are shown in Table 2. This shows that for 35 of the 38 (*i.e. *92%) these contigs, the RT-PCR data were directionally concordant with the microarray data. Further, for 27 of these 35 contigs (*i.e. *77%) the difference in gene expression measured by RT-PCR was greater than that measured by the microarrays. A corollary of this is that the reported levels of difference in gene expression determined from the microarrays (Tables 3 and 4) may be conservative. These data also make clear that microarray expression data should always be further investigated by RT-PCR analyses.

**Figure 2:**
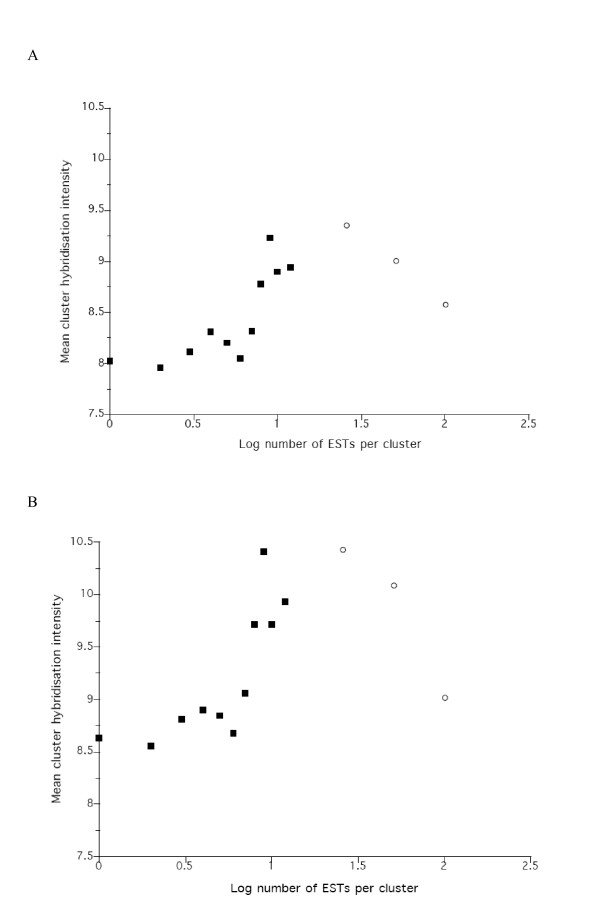
The relationship between the log number of ESTs within a cluster and the observed mean cluster hybridization intensity for A) L1 and iL3 and B) L2-direct and – indirect microarray hybridization in which clusters containing more than 10 ESTs were grouped into three categories (11–25, 26–50 and > 51, nominally set as 25, 50 and 100, respectively) (○) and one >10 category, nominally set as 11 (■)).

We investigated the relationship between the observed microarray hybridization intensity of clusters and the number of ESTs contained within the respective cluster. The rationale for this analysis, was that the number of ESTs within a cluster is commonly used as a measure of the level of gene expression; for this to be correct a positive relationship with microarray hybridization intensity should be observed. In this analysis, we treated clusters containing large numbers of ESTs in two ways such that clusters containing more than 10 ESTs were grouped into (i) three categories; 11–25, 26–50, > 51 or (ii) one category; > 10, ESTs per cluster. Overall, there is a positive relationship between the number of ESTs within a cluster and the observed mean cluster hybridization intensity, but that this relationship breaks down for clusters that contain more than *c*. 20 ESTs (Figure 2A). There are two, non-mutually exclusive reasons for this lack of correlation for very large clusters. Firstly, the large representation of the clusters on the microarray itself may have affected the hybridization kinetics of members of these large clusters. Secondly, the high EST representation of these clusters may be a quantitatively incorrect representation of the relative abundance of the mRNA abundance in the sample from which the cDNA library was constructed. Thus, a bias in the cloning and, or sequencing *etc*. of certain gene transcripts may have, at least in part, generated the high EST representation. If this latter scenario is correct, then caution should be exercised, and independent evidence generated, when using EST representation as a measure of high levels of gene expression.

Overall these analyses of gene expression of L1 and iL3s on these *S. ratti *microarrays show that robust data can be generated. A particular feature of this microarray is that component ESTs of many contigs and clusters provide replicate data (formally, quasi-replicate since the characteristics of each arrayed amplicon are not identical) for measurement of gene expression. Here we have presented data that shows a high level of concordance of measurement of gene expression between cluster and contig members. We also show that the very large representation of some clusters and contigs on the microarray does not bias observed results. The observed microarray results are, in general, confirmed by RT-PCR analysis. Comparison of measures of differences in gene expression between these two stages as determined from this *S. ratti *microarray and from analysis of EST representation in cDNA libraries [27] has shown a low concordance.

### Direct L2 v indirect L2

Gene expression was compared in free-living second stage larvae (L2) destined to develop directly into iL3 stages and in L2s destined to develop indirectly into free-living adults (Figure 1).

### Direct L2 v indirect L2: data quality assurance

30 slides were probed (*i.e*. 15 dye swap experiments) using four different biological replicates, of these 24 slides passed our quality control and went forward for analysis. Across all the biological and technical replicates, data were available for 7,476 of the 9,984 arrayed spots. 675 ESTs were identified whose expression was significantly different between the direct L2 and the indirect L2 stages (p ≤ 0.05). This number is greater than the 374 significant results that would be expected by chance (7,476 × 0.05). Of these 675, the expression of 429 was significantly greater in the indirectly developing L2s and 246 significantly greater in the directly developing L2s, of which four and one ESTs were expressed two or more-fold greater in the indirect L2 and direct L2 stages, respectively (Table 5). The hybridization of control 'spikes' 3- and 10-fold different between samples resulted in mean observed differences in expression of 3.2 and 8.3, respectively. As for the L1 and iL3 data, this suggests that the dynamic range over which the observed experimental L2 microarray data fall is generally correct, though somewhat constrained compared with the actual differences in observed expression.

To asses the reproducibility of expression data between individual arrayed ESTs within their respective contigs and clusters, for each of these 675 ESTs that were significantly differentially expressed, we also analysed the expression data for other contig and cluster members (excluding single-EST clusters) present on this microarray to which these 675 ESTs belonged. The 675 ESTs belonged to 425 contigs and 404 clusters; 153 ESTs were single-EST contigs and clusters. For the 522 (675 - 153) non-single EST clusters and contigs we determined the proportion of ESTs belonging to each contig and cluster which (i) produced hybridization data that passed the quality control threshold and (ii) the proportion of these that were also significantly different between the direct and indirect stages. A mean of 99.8% and 99.8% of ESTs of these contigs and clusters, respectively, produced hybridization data that passed the quality control threshold; a mean of 57.0% and 39.3% of the ESTs of these contigs and clusters, respectively, were significantly (p ≤ 0.05) differently expressed between the direct and indirect stages. This observed level of concordance for the contigs is similar to the L1 and iL3 data, but lower for the analysis by cluster. One possibility for this difference is that, compared with the L1 and iL3 data, the clusters used in this analysis of gene expression in L2s, may less accurately represent actual units of expression in the L2 stages. This possibility would need to be investigated directly.

To further investigate the relationship of measures of expression for contigs and their respective clusters, we identified contigs that had the greatest difference in their ratio of expression (formally, the greatest mean fold difference of component ESTs that were significantly differently expressed) between the L2 direct and indirect stages, with a two or more-fold difference in expression. This analysis resulted in just two contigs whose expression was 'up' in this manner in the indirectly developing L2 and one in the directly developing L2s (Table 6); these three contigs represent three clusters.

Of the previously identified large EST clusters [27], 28 occurred in the L2 stages. From these microarray data, 99.7% of the ESTs of these 28 clusters produced hybridization data above the quality threshold, of which 8% were significantly different between the two L2 stages. For the remaining 5,529 ESTs not within these clusters, 99.8% produced hybridization data above the quality threshold, of which 9.4% were significantly different between the two L2 stages. Therefore, as for the L1 and iL3 data, the hybridization behaviour of ESTs of highly represented clusters and all other ESTs, appears to be the same.

We used RT-PCR to confirm the microarray expression data. The mean RT-PCR expression ratios of the 45 L2 direct- or L2 indirect-up contigs together with their microarray mean contig expression ratios are shown in Table 7. This shows that for 35 of these 45 (*i.e. *78%) contigs, the RT-PCR data were directionally concordant with the microarray data. Further, for 27 of these 35 (*i.e. *77%) contigs, the difference in gene expression between the samples measured by the RT-PCR was greater than that measured by the microarrays. A corollary of this is that the reported levels of difference in gene expression determined from the microarrays (*e.g. *Table 6) may be conservative.

As for the L1 and iL3 data (above) we investigated the relationship between the observed microarray hybridization intensity of clusters and the number of ESTs contained within the respective cluster. This again showed a positive relationship, but one that breaks down for clusters that contain more than *c*. 20 ESTs (Figure 2B).

The microarray analysis of gene expression in L2 stages results in data with similar characteristics to those generated from analysis of the L1 and iL3 stages.

### L1 v iL3: biological results and interpretation

The 1,903 significantly differently expressed ESTs, represent 796 contigs and 770 clusters (69 and 62 contigs and clusters, respectively, containing an EST that is expressed two or more-fold) that were expressed at a significantly higher level in the iL3 stage. In the L1 stage, 192 contigs and 166 clusters (50 contigs and 37 clusters containing an EST that is expressed two or more-fold) were expressed at significantly higher levels (Table 1).

BLAST analysis of the 62 clusters with a greater than two-fold difference in expression in the iL3 stage, compared with the L1 stage, shows that nine have no significant alignment, 25 have a significant alignment to known *C. elegans *proteins and 17 have a significant alignment to hypothetical proteins from *C. elegans*. Nine have significant alignments to other parasitic nematode ESTs, one aligns to an *Onchocerca volvulus *FAR protein and one aligns to a hypothetical protein from a mouse. Overall the maximum and mean difference in expression was 21.9 and 3.17-fold, respectively. Further details of these data are shown in Table 3.

BLAST analysis of the 37 clusters with a greater than two-fold difference in expression in the L1 stage, compared with the iL3 stage, shows that three have no significant alignment; seven have a significant alignment to hypothetical proteins from *C. elegans *and the remaining 27 have significant alignment to known *C. elegans *proteins (Table 4). Overall the maximum and mean difference in expression was 3.65 and 2.32-fold, respectively.

We used gene ontology (GO) slim analysis of a representative sequence from each cluster [27] to further understand the difference in the L1 and iL3 transcription profile. Of the 37 L1-up (two or more fold) clusters (Tables 1 and 4) approximately half the clusters could not be assigned to a process, function or component (38, 65 and 43%, respectively), but the largest remaining categories were consistent with larval growth. Thus, by GO process, of the 62% that had an assignment, 61% were classified as being involved with protein biosynthesis and metabolism, 26% with embryogenesis; by GO function, of the 35% that had an assignment, 69% were classified as being a ribosomal component; by GO component, of the 57% that had an assignment, 76% were classified as being a component of the ribosome.

A similar pattern is also seen by BLAST analysis of a representative contig consensus sequence from each cluster (Table 4). Among these 37 L1-up clusters, 60% have significant alignment to ribosomal or ribosomal-associated proteins and 13.5% align to other proteins known to have housekeeping functions or functions required for larval growth (*e.g*. proton transport and cuticle synthesis). In addition, 19% align to hypothetical proteins and 7.5% have no significant alignment. This view of gene expression in the L1 stage is consistent with these stages undertaking substantial protein synthesis, consistent with growth of these stages as L1s and in preparation for development to the L2 stage (Figure 1).

The picture for iL3 stages is less clear. GO slim analysis of the 62 iL3-up (two or more fold) clusters (Tables 1 and 3) showed that most clusters (71, 79 and 77%, respectively) could not be assigned to a process, function or component. The remainder of the classifications belonged to a diversity of processes, functions or components with no clear, unifying theme. BLAST analysis of these 62 clusters, using a representative contig consensus sequence, showed that 14.5% have no significant alignment, 29% align to hypothetical proteins and 14.5% align to other nematode ESTs; there are various other alignments (42%) to known *C. elegans *proteins but, again, with no unifying theme; there are no alignments to ribosomal proteins. Overall, this shows that the gene expression of the iL3 stage is very different to that of the L1 stage. This result is, therefore, consistent with the iL3 stage not growing, as is obvious from observation of its biology. The high proportion of iL3-up genes with significant alignment to hypothetical proteins, or to uncharacterised ESTs, or with no significant alignment at all, suggests that the transcriptional profile of these infective stages involves genes whose role and function remain to be elucidated in nematodes (including *C. elegans*) and other organisms. More broadly this suggests that genes involved in the biology of the infective stage of *S. ratti*, and perhaps other parasitic nematodes, remains to be investigated. These infective stages are a crucial step in the transmission of parasitic nematodes. It can be envisaged that approaches to the control of parasitic nematodes could be targeted against such infective stages. Discovering genes crucial to the biology of infective larvae could be important in this respect.

We investigated the relationship of the *S. ratti *genes whose expression was different between L1 and iL3 stages, to genes of *C. elegans*. In *C. elegans*, genes have been identified that when knocked-down by RNA interference (RNAi) result in defects in growth, larval arrest, morphology, movement or reproduction [36]. For each of the 659 L1-up and 1,244 iL3-up ESTs (Table 1) we identified a *C. elegans *gene with these RNAi phenotypes. This was done by matching the most significant BLAST alignment (directed against *C. elegans *only, [27]) of the 1,903 *S. ratti *ESTs to the *C. elegans *genes with these RNAi phenotypes. Overall, 1,622 of these *S. ratti *ESTs had such alignments; 281 did not. We then calculated the mean L1 : iL3 fold difference in expression of these *S. ratti *ESTs that (i) had a significant BLAST alignment and (ii) did not have a significant BLAST alignment with the *C. elegans *genes, and compared these using a t-test (Table 8). This shows that there is a significant difference in the expression of these *S. ratti *ESTs between L1 and iL3 stages for each of the *C. elegans *RNAi phenotypic classes, with greater average expression in L1 stages compared with iL3s (Table 8). Thus, *S. ratti *genes whose expression is, on average, greater in L1s than in iL3s are more likely to have an alignment to *C. elegans *genes which themselves have, at least one, of these RNAi phenotypes. There are two, mutually non-exclusive reasons for this observation. Firstly, the role of these *S. ratti *L1-up genes may be more central to core aspects of nematode biology, such that they are, comparatively, over represented in the *C. elegans *RNAi phenotypic classes. Secondly, the *C. elegans *RNAi analysis may have under-reported genes with phenotypes present in, or relevant to, dauer larvae (the likely analogues of iL3s of parasitic nematodes [13]). This second possibility further suggests that the genetic basis of the biology of infective L3s of *S. ratti *and other parasitic nematodes is under explored.

Analogously lists of genes identified by SAGE as being differentially expressed in *C. elegans *dauer larvae compared with non-dauer, mixed life-cycle stages were obtained [5,37,38] and compared (as above) with the *S. ratti *L1-up and iL3s-up ESTs. This analysis identified 332 matches between the *S. ratti *ESTs and the *C. elegans *gene lists. For each of these 332 genes and ESTs, the *C. elegans *dauer/non-dauer ratio was plotted against the *S. ratti *L1 : iL3 ratio. No correlation was observed (data not shown), which suggests that there is little identifiable transcriptional conservation in these life-cycle stages between *S. ratti *and *C. elegans*. Similarly, for genes matched between *S. ratti *and *C. elegans *there was no correlation between the *S. ratti *mean intensity of expression and the *C. elegans *SAGE measures of intensity of expression (data not shown).

We also investigated the relationship of L1 and iL3 gene expression in *S. ratti *and *S. stercoralis*. For *S. stercoralis*, contigs have been identified (by EST representation analysis) whose expression is potentially L1- or iL3- specific or -biased [[17,39], Mitreva pers. comm.]. Specifically, 207 and 168 contigs were identified as being L1- and iL3-biased, respectively and 1,362 and 1,616 contigs as being L1- and iL3-specific, respectively in *S. stercoralis*. To compare these putatively differently expressed *S. stercoralis *contigs with the expression of *S. ratti *contigs, measured by microarray analysis, we identified matching *S. ratti *and *S. stercoralis *contigs by local BLAST alignment of representative *S. stercoralis *contig consensus sequences to *S. ratti *representative contig consensus sequences. All 988 *S. ratti *contigs (Table 1) had a significant *S. stercoralis *alignment. We then determined the mean L1 : iL3 expression ratio of these *S. ratti *contigs that had such matches to the *S. stercoralis *contigs in each of the four categories of *S. stercoralis *expression and those that did not, and compared these using a t-test (Table 9). This analysis shows that the *S. ratti *alignments of *S. stercoralis *L1-biased contigs were significantly differently expressed in *S. ratti*, but with greater expression in iL3 stages, compared with L1s. There are further significant differences in the expression of *S. ratti *genes with respect to the *S. stercoralis *contigs though this difference is in the magnitude of the difference in expression in iL3s rather than between iL3s and L1s (Table 9). In all, these results suggest that there is little conservation of L1- and iL3 biased and -specific gene expression of *S. ratti *(measured by microarrays) and of *S. stercoralis *(measured by representation of ESTs in staged libraries).

The different methods used to measure gene expression in *S. ratti, S. stercoralis *and *C. elegans *may be a source of significant differences in the observed transcriptional profile. In other systems, comparison of SAGE and microarray [40] and EST representation and microarray [41] data have shown rather limited concordance; comparison of six different microarray platforms found substantial similarity in the observed expression [42]. For the parasitic nematode *Haemonchus contortus*, comparison between SAGE and two EST libraries showed some, though not extensive, similarity in expression for specific genes, though when analysed by functional classes, rather greater similarity was apparent [43]. During the construction of cDNA libraries, biases in the representation of cDNA species can occur due to several factors that affect the cDNA synthesis at various steps. For example the AT content of the starting material or the secondary structure of the different RNA molecules can inhibit the function of reverse transcriptase. Large transcripts are often prematurely truncated due to the inefficiencies of reverse transcriptase and size fractionation methods for cDNA purification can exclude some transcripts. In some cases, following cloning, inserts are lost due to vector-driven expression of deleterious genes, vector instability following insert driven transcription and finally inappropriate antibiotic resistance. Furthermore, it is unusual for technical and biological repeats to be used in construction of such libraries which has the consequence that any EST representation analysis is based on a statistical sample size of one. This is in contrast to SAGE and microarray analyses which have replicate measures, so as to facilitate rigorous statistical analyses [44,45].

Comparison of gene expression between L1 and iL3 stages has shown that there are extensive differences. Genes with significantly greater expression in L1 are largely concerned with growth. The identity and likely role of those with significantly greater expression in iL3s are unknown. There is little evidence of observed commonality of transcription between L1 and iL3s of *S. ratti *and *S. stercoralis*, nor between *S. ratti *and *C. elegans *dauer larvae.

### L2 direct v L2 indirect: biological results and interpretation

The 675 significantly differently expressed ESTs, represent 105 contigs and 93 clusters (one contig and cluster, containing an EST whose expression was different by two or more-fold) that were expressed at a significantly higher level in the directly developing L2s. For indirectly developing L2s, 320 contigs and 311 clusters (two contigs and clusters containing an EST whose expression was different by two or more-fold) were expressed at significantly higher levels (Table 5). Blast analysis of these clusters whose expression was two or more-fold greater is shown in Table 6. The cluster from the L2-direct stage has a significant alignment to a *C. elegans *hypothetical protein, and the two clusters from the L2-indirect stage have significant alignments to a *C. elegans *probable peroxiredoxin and a *Schistosoma mansoni *ORF. There were no gene ontology (GO) slim designations for these clusters.

In the L2 indirect-up category, 16.2% (95% CI 12.4–20.5) of contigs align to hypothetical and unknown genes whereas in the L2 direct-up contigs there are 8.57% (95% CI 6.36–12.64). The L2 library contains 14.5% (95% CI 12.9–17.3) contigs that align to unknown genes [27]. Thus, genes whose expression is comparatively greater in directly developing L2s are less likely to be previously uncharacterised genes, compared with all L2 ESTs and compared with those genes with significantly greater expression in the directly developing L2s. Directly developing L2s develop into iL3s and there is therefore a contrast between the two analyses undertaken here. Comparative analysis of L1 and iL3 gene expression (above), suggests that gene expression in iL3s is of, comparatively, unknown genes, whereas this analysis (above) suggests a contrary situation for the preceding L2 stage.

Analysis of gene expression in L2 stages with different developmental fates only reveals small, but significant, differences in gene expression.

## Conclusion

This first study of multi-gene expression in the parasitic nematode *S. ratti *using a microarray of ESTs has shown that robust data can be generated; that measures of expression derived from within multi-EST contigs and clusters are consistent and that there are unlikely to be any biases in observed results due to EST-dense clusters. The observed microarray expression data support overall the computationally determined clusters and contigs as units of expression. Comparison of EST representation and microarray data show substantial inconsistencies in identification of (i) highly abundant expressed genes and (ii) life-cycle stage-specific or -biased expression. The observed microarray results are, in general, supported by gene specific RT-PCR data. We find little evidence for the conservation of transcriptional profiles between *S. ratti *(as measured by microarrays) and *S. stercoralis *(as measured by EST abundance); nor between *S. ratti *and *C. elegans*. Part of this difference is likely to be due to the different approaches used in the measurement of gene expression. This difference is likely to be exacerbated by the observation of the substantial inter-specific differences in the discovered expressed genes [25].

Within the *S. ratti *life-cycle we find that the biologically distinct L1 and iL3 stages are similarly transcriptionally distinct. The predominant role of genes more highly expressed in L1 stages is involvement with protein synthesis and thus growth, commensurate with their observed biology. Genes more highly expressed in iL3 stages are, comparatively, less easy to assign a role or function to them. More generally, it is significant that many of the genes whose expression is significantly different between L1 and iL3 stages are unknown beyond alignments to proteins predicted from the *C. elegans *genome sequence. This highlights the substantial experimental challenge that lies ahead in actually determining the role and function of these genes in the *S. ratti *lifecycle. The analysis of L1 and iL3 gene expression has, though, identified genes likely to be specifically important in the biology of these stages, which can therefore direct future effort at discovering the role and function of these genes. The transcriptional profiles of L2 stages with different (*i.e. *direct and indirect) future development are very similar, though not identical.

## Methods

### Parasite material

The *S. ratti *isofemale lines ED321 Heterogonic and ED5 Homogonic were used in this study [7]. Both isofemale lines were maintained in female Wistar rats and infections were initiated with 1,000 iL3s and free-living stages grown in faecal cultures at 19°C, unless otherwise stated, all as previously described [7,46]. Free-living material was prepared, cleaned and RNA isolated as described elsewhere [19,27]. For all worm preparations (below), worms were concentrated into a volume of 200 μl to which an equal volume of TRI reagent (Sigma Genosys Ltd., UK) was added, which was then snap frozen in liquid nitrogen and subsequently stored at -80°C until required. Two different analyses of gene expression in the free-living phase of the *S. ratti *life-cycle were undertaken.

#### i. L1 v iL3

Isofemale line ED321 Heterogonic was used. Fresh faeces were collected from *S. ratti*-infected rats at days 5, 6, 7 and 8 p.i. and L1s prepared with a Baermann funnel held for 6 h at 19°C. The larvae were concentrated by centrifugation and then cleaned by flotation on 60% v/v sucrose, as previously described [19,27]. Infective L3s were harvested from 14 day-old faecal cultures [19,27] that had been maintained at 19°C. The iL3s were cleaned by sucrose flotation as for the L1s, above. In excess of 150,000 larvae of either stage were routinely isolated from 6 infected hosts.

#### ii. L2 direct v L2 indirect

*S. ratti *isofemale line ED321 Heterogonic predominantly undergoes indirect development; isofemale line ED5 Homogonic predominantly undergoes direct development [7]. Therefore, L2 stages of ED321 Heterogonic and of ED5 Homogonic are destined for indirect (*i.e. *L2 indirect) and direct (*i.e. *L2 direct) development, respectively; these sources of material were used in this comparison. To do this, for both isofemale lines, rats were infected with ED321 Heterogonic or ED5 Homogonic and faeces collected on days 5, 6, 7 and 8 p.i. and cultured for 24 h at 19°C, after which larvae were prepared with a Baermann funnel held for 6 h at 19°C, larvae were concentrated and cleaned by sucrose flotation, as above. In excess of 75,000 worms were routinely isolated from three infected hosts.

For both these comparisons, the experimental design used was to have at least three biological replicates for each sample (*i.e. *three independent preparations of the relevant worm samples and their RNA) and to have at least three technical replicates (*i.e. *independent, separate cDNA synthesis, amplification and hybridization *etc.*) for each biological replicate. For each hybridization (below) adye-swap was used *i.e. *each sample to be used in a hybridization was labelled, separately, with each of the two dyes (below).

### Microarray production

21,085 ESTs were sequenced from various *S. ratti *stage-specific libraries, of which 14,761 resulted in sequence data above a quality threshold that were then submitted to public databases [27]. 9,534 clones were derived from the *S. ratti *free-living libraries of which 7,182 produced sequence data (above a quality threshold); however, all of these 9,534 clones were used to construct the microarray. The three libraries of free-living stages were L1, L2 and mixed iL3/free-living adults. The material used for the construction of this latter library was 54% iL3s, 26% free-living females, 18% free-living males and 2% L2s. These 7,182 ESTs are highly redundant, since they represent 2,742 contigs and 2,590 clusters, both including 1,523 singletons. Notwithstanding this redundancy, the 9,534 clones were used in the microarray construction for two reasons: (i) this approach was less error-prone than attempting to select a unique clone set and (ii) this in-built redundancy provides many replicates of individual contigs and clusters, which can be exploited in quality-control analyses. 308 ESTs from the day 6 p.i. parasitic female library (representing 260 contigs and 255 clusters, 105 of which are singletons, *i.e. *single EST clusters and contigs) were included as an internal control, to ensure that gene expression in the parasitic and free-living stages could be differentiated.

Cloned ESTs were obtained from the Genome Sequencing Centre (Washington University, MO, USA) as glycerol stocks in a 384-well format. Replicates of these stocks were grown overnight in 384 deep-well plates (Greiner Bio-One Ltd., UK) containing 50 μl L-broth with 50 μg/ml ampicillin at 37°C in an orbital shaker. Insert-spanning PCR was performed (still in 384-well format; MJ Research Inc., USA) in a 20 μl volume, consisting of: 1 μl fresh overnight culture; 0.25 mM of each 5'-amino-C6-labelled Sp6/T7 or TopoFor/TopoRev vector primers [see Additional file 3] (Sigma Genosys Ltd., UK); 0.2 mM each dNTP (Pharmacia); 1× HotStarTaq PCR Buffer; 0.5 units HotStarTaq (both Qiagen Ltd., UK), with amplification conditions of 95°C for 15 min; 30 cycles of 94°C 45 s, 55°C 45 s, 72°C 1 min with a final extension at 72°C for 7 min. All amplicons were visualised on standard 1% w/v agarose gels stained with ethidium bromide to determine the PCR success rate (results not shown). For any plates with less than 75% success, amplification of the whole plate was repeated.

The resulting amplicons were precipitated with a final concentration of 300 mM sodium acetate (pH 5.2) and two volumes of 100% ethanol at -70°C for three hours, after which they were centrifuged at 4°C for 30 min at 1600 **g**, the supernatants discarded and the pellets washed twice with 70% v/v ethanol and air dried before finally being resuspended in 25 μl microarray spotting buffer (50 mM sodium phosphate buffer, pH 8.5), giving a final DNA concentration greater than 0.1 mg/ml.

Duplicate-spotted arrays of these PCR fragments were printed in blocks of 20 × 21 spots, with three fields each of 16 blocks. There were four blank spots in each block to allow array orientation [47]; thus, each cloned EST was present on each microarray slide as a pair of spots. The DNA was spotted onto CodeLink activated microarray slides (Amersham Biosciences UK, Ltd.); these slides are coated with a hydrophilic polymer containing N-hydroxysuccinimide ester reactive groups that can covalently immobilize amine-modified DNA. Slides were printed in a relative humidity of < 50% using a BioRobotics Microgrid II arrayer (Genomic Solutions Ltd., UK) with MicroSpot 2500 split pins, as instructed by the manufacturer. Immediately after spotting, the DNA was coupled to the printed slides by incubating in an airtight container, above a saturated sodium chloride solution, for 24 h at room temperature. Residual reactive groups on the slides were blocked in 0.1 M Tris, 50 mM ethanolamine, pH 9.0 at 50°C for 30 min, briefly washed twice in distilled water then incubated for 30 min in 4 × SSC, 0.1% w/v SDS at 50°C, with shaking, briefly washed twice in distilled water, prior to a 30 s incubation in water at 100°C, briefly washed twice in distilled water, all as described in the manufacturer's protocol. The slides were dried by centrifugation at room temperature for three minutes at 120 **g**, and then stored at room temperature in a dust free container with a desiccated environment until use.

### Microarray controls

A number of controls were included on the microarray. Firstly, the controls from the Lucidea Universal Scorecard (Amersham Biosciences UK, Ltd.) were included. These consist of 10 calibration controls, 8 ratio controls, 3 utility controls and 2 negative controls. The calibration controls (which are detected using 'spikes' of different concentrations of probe that are added to the hybridization solution, see below) measures the sensitivity and dynamic range of the hybridization reaction, over 4.5 orders of magnitude. The ratio controls (which are detected using 'spikes' of different ratios of probe between the two hybridization samples under comparison, see below) measure different ratios of gene expression, over a range of 2.5 orders of magnitude. The negative controls determine if non-specific hybridization is occurring. All these controls were resuspended in spotting buffer to a final concentration of 50 ng/ml and spotted in duplicate, four times on each microarray slide. In addition, further negative controls of (i) an amine-linked poly-A fragment (0.1 mg/ml) in 1 × spotting buffer [47] and (ii) 1 × microarray spotting buffer alone were included, which were used to monitor any non-specific hybridization. In summary each slide contained the controls: 92 (each spotted in duplicate to give 184 spots) Lucidea Universal Scorecard controls, 308 (616 spots in total) amplicons from the parasitic female libraries, 5 (10 spots in total) poly-A fragments, and 45 (90 spots in total) spotting-buffer only; the remainder of the slide contained 9,534 (19,068) amplicons from the free-living libraries (see *Microarray production*, above). For all analyses, the poly-A and spotting buffer only control spots resulted in a very low signal, representing non-specific background hybridization. These spot values were used to calculate background levels for subtraction (below).

### Probe generation and microarray hybridizations

Pilot experiments showed that the quantity of RNA and resultant cDNA that could be produced from some of our samples was at, or below, the quantity required for successful, robust hybridization and appropriate repeats (data not shown). To overcome this, a single round of cDNA amplification was used in all subsequent hybridizations. Microarray slides were hybridized in reverse dye-swap experiments using aminoallyl labelled cDNA probes generated from amplified total RNA samples that were conjugated to Alexa Fluor dyes 555 (green) and 647 (red) (Molecular Probes Inc., USA). To do this, total RNA was extracted from the appropriate larval stages using TRI reagent (Sigma Genosys Ltd., UK) as previously described [19,27]. 1–5 μg of total RNA was spiked with the relevant Lucidea Universal Scorecard controls (Amersham Biosciences UK, Ltd.). These spiked samples were amplified in the presence of 5-(3-aminoallyl)-2'-deoxyuridine 5'-triphosphate (AA-dUTP) using the Amino Allyl MessageAmp aRNA kit (Ambion (Europe) Ltd.) exactly as described by the manufacturers. 100 μg of the resulting aminoallyl-labelled cDNA (AA-cDNA) was divided equally for esterification to each of the two (red or green) Alexa Fluor dyes (Molecular Probes Inc., USA) following the manufacturer's instructions. The labelled AA-cDNA was purified on MinElute PCR Purification columns (Qiagen Ltd., UK) following the manufacturer's instructions, except that the wash step was replaced with three washes with 750 μl of 75% v/v ethanol and a final elution with 11 μl of distilled water.

For hybridization, the resulting red (Sample A) or green (Sample B) dye-labelled AA-cDNA samples were mixed (9 μl of each) and added to an equal volume of 2 × GenHYB hybridization buffer (Genetix Ltd., UK) containing 1 μg/μl Poly dA (Amersham Pharmacia Biotech, Ltd., UK). In the reverse dye-swap hybridization the green labelled (Sample A) and the red labelled (Sample B) were used. The microarrays were hybridized overnight, at 42°C, in a final volume of 40 μl under 22 mm × 60 mm plastic Hybri-slips (Sigma-Aldrich Company Ltd., UK) inside hybridization chambers (Camlab Ltd., UK) containing 3 × SSC in each of the chamber's reservoirs. After hybridization, slides were subjected to successive washes; firstly 5 min, 2 × SSC, 0.1% w/v SDS at 42°C and then remaining washes, all at room temperature; 5 min, 1 × SSC, 0.1% w/v SDS; 1 min, 0.2 × SSC, 0.1% w/v SDS; 1 min, distilled water; 1 min, 100% ethanol. Slides were dried immediately by centrifugation at 200 **g **for 5 min at room temperature. The resulting hybridized slides were scanned with an Axon Instruments GenePix 4000 B dual laser microarray scanner with integrated GenePix software for image acquisition.

### Data and statistical analyses

For both the L1 v iL3s and the L2 direct v L2 indirect comparison, we sought amplicons whose expression were significantly different between the samples. GenePix format file data from the scanned slides were imported using a custom PERL script [48] and all subsequent analyses were performed with custom PERL scripts and system calls to the statistical programming language R [49]. Only microarray features flagged as 'good' by GenePix were imported. The samples under consideration (L1 v iL3 and L2 direct v L2 indirect) were given arbitrary designations of 'signal' and 'control'. For each slide, the background level of hybridization was calculated as the median value of the negative control spots (poly-A fragments and spotting-buffer, above) for the signal and for the control samples separately, and these background values were subtracted from the signal and from the control values of each spot on the microarray, respectively. For any resultant hybridization values of less than 10, this was re-set to 10.

The log_2_(signal/control ratio), M, and the log_2_(mean of the signal and control intensity), A, for each spot on each slide was calculated. These data were processed using the loess function in R, which corrects for intensity-dependant non-linearity in M [50]. A scatter plot of M against A was created for each slide and those slides judged visually to have normalised poorly (*i.e. *non-linear relationship between M and A) or with significantly lower than average intensities (*i.e. *poor hybridization) were discarded. The microarray slides that survived this process were used in subsequent statistical analyses.

Loess normalised technical-replicate data (*i.e. *data for each spot on each slide, including dye swaps) were used to calculate a mean value of M for each amplicon present on the microarray for each biological replicate. From this, a paired t-test was applied to M, with n-1 degrees of freedom, where n was the number of biological replicates available for the feature being investigated, with the null hypothesis that there was no significant difference between the signal and control values. For each EST critical two-tailed values of t for each degree of freedom and p values were obtained from [51]. In these analyses, large numbers of t-tests were undertaken such that some of the identified statistically significant ESTs will be due to chance alone (*i.e. *type I errors). However, we favour using these microarray data as primary screens of gene expression followed by subsequent confirmation by gene-specific RT-PCR studies. Contig and cluster membership were assigned to each EST feature. The mean ratio was then calculated for each contig and cluster using only the constituent ESTs that were themselves significantly different. The fold difference for each EST, contig or cluster were then calculated from these log ratios; these are reported here.

The microarray experiments included in this manuscript have been submitted to ArrayExpress: accession numbers E-MEXP-697 and E-MEXP-709.

### Reverse transcriptase-PCR

Total RNA samples were extracted using the TRI reagent method (see above). cDNA synthesis (and genomic DNA elimination) was carried out for each sample using 0.5 μg total RNA. To do this the Quantitect reverse transcription kit (Qiagen Ltd., UK) was used exactly as described in the manufacturer's protocol. PCR reactions were carried out using 0.5 μl of neat cDNA or 0.5 μl of control cDNA (*i.e. *the 'synthesis' of cDNA samples in the absence of any reverse transcriptase, which will therefore monitor for contamination with genomic DNA). The reactions were performed using 1.1 × PCR Master Mix (ABgene, Advanced Biotechnologies Ltd., UK) containing 1.25 units *Taq *DNA polymerase, 75 mM Tris-HCl, 20 mM ammonium sulphate, 1.5 mM MgCl_2_, 0.01% v/v Tween 20, 0.2 mM each dNTP, 1 μM each primer (test pair or control pair) and distilled water to achieve a final volume of 20 μl in a 96 well format. Cycling conditions were: 94°C 3 min; 27 (L1 v iL3) or 29 (L2) cycles of 94°C 1 min, 55°C 1 min, 72°C 1 min; 72°C 5 min. 30 cycle PCR reactions were also performed on each batch of cDNA and control cDNA using at least three control primer pairs to check for contamination with genomic DNA. If the results of this were consistent with the absence of contamination with genomic DNA then the cDNA sample was used experimentally. Loading dye was added to each sample and 10 μl were loaded onto 2% w/v TAE agarose gels stained with ethidium bromide, together with molecular weight markers of known concentration (ABgene, Advanced Biotechnologies Ltd., UK). Gel images were captured using the image acquisition software Gene Snap (Syngene Ltd., UK) on a Gene Genius Bio Imaging System (Syngene Ltd., UK) and the concentration of DNA in each amplified band was estimated using the GeneTools (Syngene Ltd., UK) analysis programme against the known molecular weight standards included on the gels. For each experimental series (*i.e. *L1 and iL3, and L2 direct and L2 indirect) the amplification of two control genes was used to confirm that equal amounts of cDNA were present in the respective samples under comparison. These were contigs SR00785 and SR00113 for the L1 and iL3 comparison and SR00015 and SR00113 for the L2 direct and L2 indirect comparison; SR00113, as *act-3*, has previously been successfully used in this role with *S. ratti *[19,27]. Primers pairs were designed using contig consensus sequences and used to amplify a selection of genes that showed a range of different expression levels between the two stages being tested. Thirty-eight L1- or iL3-up contigs and 45 L2 direct- or L2 indirect-up contigs were analysed by RT-PCR using 27 and 29 amplification cycles, respectively, which was the lowest cycle number at which amplification could be visualised. Two and three biological replicates, each with at least two technical replicates, were used for L1- or iL3-up contigs and the L2 direct- or L2 indirect-up contigs, respectively. The mean L1 : iL3 and mean L2 direct : L2 indirect ratio was the average of each of the biological replicates which were themselves the mean of the technical replicates within each biological replicate. If no amplification of either sample occurred, no data were recorded. If there was no detectable amplification for one sample, then the resulting intensity value was set to a value of one.

## Abbreviations

BLAST, basic local alignment search tool; EST, expressed sequence tag; iL3, infective third stage larvae; GO, Gene Ontology; L, larval stage; ORF, open reading frame; p.i., post infection; RNAi, RNA interference; SAGE, serial analysis of gene expression.

## Authors' contributions

FJT generated the material for the microarrays, performed the hybridizations, collected the data, co-ordinated the project and co-drafted the manuscript; GLAB contributed to the experimental design and performed all the statistical analyses; LH carried out the RT-PCR experiments and produced nematode material; CPW contributed to the microarray production and produced nematode material; JC printed the microarrays and provided technical support throughout; MEV conceived and directed the study and analyses, analysed the RT-PCR data and co-drafted the manuscript. All of the authors read and approved the final manuscript.

## Supplementary Material

Additional file 1 A table detailing the L1-up contigs and clusters. Click here for file

Additional file 2 A table detailing the iL3-up contigs and clusters. Click here for file

Additional file 3 A table detailing PCR primer sequences. Click here for file

## Figures and Tables

**Table 1:** The number of ESTs, contigs and clusters differently expressed in L1 and iL3 stages and those with a two-fold or greater difference in expression.

	**L1-up**	**iL3-up**	**Total**
**EST**^**1**^	659	1,244	1,903
**≥ 2-fold**^**2**^	217	147	364
**Contig**^**3**^	192	796	988
**≥ 2-fold**^**4**^	50	69	119
**Cluster**^**3**^	166	770	936
**≥ 2-fold**^**4**^	37	62	99

^1^These ESTs are represented by 203 and 178 (L1-up) and 817 and 793 (iL3-up) contigs and clusters respectively.

^2^Number of ESTs that have a ratio of expression that is ≥ 2; these ESTs are represented by 72 and 60 (L1-up) and 82 and 75 (iL3-up) contigs and clusters respectively.

^3 ^Number of contigs or clusters, respectively, in which at least one EST member (for which there were data above the quality threshold) had a significantly different ratio of expression.

^4^Number of contigs or clusters, respectively, in which at least one EST member (for which there were data above the quality threshold) has a significantly different ratio of expression that is ≥ 2.

**Table 2:** Reverse transcriptase-PCR analysis of the expression of clusters and contigs differentially expressed between the L1 and iL3s stages.

**Cluster**^**1**^	**Fold**^**1**^	**Contig**^**1**^	**Fold**^**1**^	**RT-PCR mean (SD)**^**2**^
SR00369	3.6	SR01101	3.89	0.84 (0)
SR00073	2.46	SR01013	2.46	1.02 (0)
SR00647	3.68	SR00647	3.68	3.13 (3.7)
SR00369	3.6	SR01099	3.1	4.34 (4.2)
SR00001	2.83	SR00001	5.82	4.18 (0.2)
SR00369	3.6	SR01100	5.82	1.7 (1.0)
SR00762	3.2	SR00762	3.2	18.7 (0.14)
SR00369	3.6	SR00853	4.24	5.74 (6.6)
SR00222	4.46	SR00222	4.45	18.3 (5.8)
SR03050	3.51	SR03050	3.51	2.69 (2.3)
SR01720	2.93	SR01720	2.93	9.7 (3.9)
SR00448	2.60	SR00448	2.59	9.6 (12.5)
SR01335	2.22	SR01335	2.22	3.63 (4.0)
SR00906	2.06	SR00906	2.06	3.39 (2.8)
SR02481	1.85	SR02481	1.85	7.61 (0.71)
SR01791	1.64	SR01791	1.64	2.4 (1.3)
SR00681	1.52	SR00681	1.52	4.3 (3.7)
SR01787	1.42	SR01787	1.42	2.72 (3.7)
SR00322	1.16	SR00322	1.16	8.08 (9.6)
**SR00026**	**2.06**	**SR00172**	**2.88**	**6.71 (0)**
**SR00026**	**2.06**	**SR00047**	**2.33**	**1.77 (0.22)**
**SR00012**	**2.27**	**SR01022**	**2.74**	**0.12 (0)**
**SR01012**	**2.5**	**SR01012**	**2.5**	**6.5 (0)**
**SR00946**	**1.96**	**SR01050**	**2.02**	**8.75 (10.3)**
**SR00012**	**2.27**	**SR00685**	**3.1**	**15.9 (20.1)**
**SR01053**	**1.32**	**SR01053**	**1.32**	**2.44 (2.9)**
**SR00880**	**3.13**	**SR00880**	**3.13**	**5.63 (6.9)**
**SR00978**	**2.2**	**SR00978**	**2.2**	**2.86 (2.8)**
**SR01196**	**3.65**	**SR01196**	**3.66**	**10 (8.5)**
**SR02281**	**2**	**SR02281**	**2**	**3.22 (3.2)**
**SR00930**	**1.87**	**SR00930**	**1.87**	**1.04 (0.31)**
**SR01051**	**1.71**	**SR01051**	**1.71**	**1.18 (0.9)**
**SR00957**	**1.44**	**SR00957**	**1.44**	**3.41 (1.9)**
**SR02851**	**1.43**	**SR02851**	**1.43**	**1.45 (0.32)**
**SR00194**	**1.42**	**SR00194**	**1.42**	**0.28 (0.39)**
**SR00084**	**1.39**	**SR01021**	**1.39**	**6.34 (5.5)**
**SR00795**	**1.2**	**SR00795**	**1.2**	**3.97 (0)**
**SR00230**	**1.11**	**SR00230**	**1.11**	**5.39 (2.7)**
SR00163*	1.91	SR00785	3.12	0.89 (0.12)
SR00113*	1.54	SR00113	-	0.99 (0.15)

^1^Mean cluster and contig fold difference in expression between L1 and iL3 stages measured by microarrays for iL3-up (normal text) and L1-up (bold text) clusters and contigs and for reference genes (*); – indicates that there were no significant data generated for this contig.

^2^The mean (SD) fold difference in expression as measured by RT-PCR is calculated as iL3s/L1 for iL3-up (normal text) contigs and clusters and L1/iL3 for L1-up (bold text) clusters and contigs and for the reference genes (*).

**Table 3:** The 62 iL3-up clusters (Table 1) showing the mean cluster fold change between iL3 and L1 stages, the most significant BLAST alignment and the GO slim terms, arranged by descending iL3 : L1 difference in expression.

**Cluster**	**Expression**^**1**^	**BLAST alignment**^**2**^	**E**^**2**^	**Accession number**^**2**^	**Gene ontology (GO) slim term**^**3**^		
					
					**Process**	**Function**	**Component**
SR00272	21.86	-^4^	-	-	-^4^	-	-
SR02119	9.45	-	-	-	-	-	-
SR01748	4.66	*Ce*^5 ^hypo prot^6^: predicted	1.00E-13	Y25C1A.3	-	-	-
SR00222	4.46	-	-	-	-	-	-
SR02762	4.29	*Ce *hypo prot: confirmed	5.00E-14	B0213.17	-	-	-
SR00107	3.97	*Ce *ITR-1	4.00E-28	F33D4.2f	rhythmic behaviour, muscle contraction, defecation	inositol-1-,4-,5-triphosphate receptor activity	membrane, endoplasmic reticulum
SR01733	3.78	-	-	-	-	-	-
SR00647	3.68	*Ce *PAT-10	1.00E-79	F54C1.7	-	calcium ion binding	-
SR02628	3.63	*Ce *tyrosine aminotransferase	6.00E-46	F42D1.2	L-phenylalanine catabolism, tyrosine catabolism	tyrosine transaminase activity	unknown
SR00369	3.60	*Ce *LGG-1	1.00E-58	C32D5.9	protein targeting	tubulin binding	plasma membrane
SR03050	3.51	*Ss *EST	9.00E-28	TBN95TM-SSR	-	-	-
SR01141	3.46	*Ce *TTH-1	2.00E-25	F08F1.8	-	-	-
SR01368	3.34	*Ce *hypo prot: partially confirmed	2.00E-11	W06A7.3e	-	unknown	-
SR01871	3.29	*Ce *VAP-1	6.00E-08	F11C7.3b	unknown	unknown	endoplasmic reticulum
SR00762	3.20	*Ce *hypo prot: partially confirmed	5.00E-81	ZC8.4b	-	motor activity	non-muscle myosin
SR01845	3.18	*Mm *hypo prot	1.00E-08	XP_358683	-	-	-
SR03037	3.16	*Ce *transthyretin-like family	3.00E-41	C40H1.5	-	unknown	-
SR01142	3.05	*Ce *LPD-5	9.00E-61	ZK973.10	embryogenesis and morphogenesis, larval development	-	-
SR02353	3.05	*Ss *cDNA	2.00E-05	TBN95TM-SSFH	-	-	-
SR00386	3.00	-	-	-	-	-	-
SR01720	2.93	*Ce *FRM-1	2.00E-17	ZK270.2d	-	-	-
SR01943	2.87	*Pt *EST	4.00E-17	NK^7^	-	-	-
SR00001	2.83	*Ss EST*	2.00E-10	TBN95TM-SSR	-	-	-
SR02086	2.77	*Pt *EST	6.00E-11	NK	-	-	-
SR00424	2.73	-	-	-	-	-	-
SR02886	2.71	*Ce *hypo prot: confirmed	3.00E-74	ZK1073.1	spermatogenesis, cell differentiation, negative regulation of cell growth	unknown	-
SR02990	2.68	*Ov *FAR-1	3.00E-15	F02A9.2	-	-	-
SR03016	2.64	*Ce *hypo prot: predicted	9.00E-05	R07H5.10	-	-	-
SR00448	2.60	*Ce *SKR-1	3.00E-63	F46A9.5	embryogenesis and morphogenesis, reproduction	-	-
SR00092	2.53	*Ce *hypo prot: partially confirmed	3.00E-08	F55F8.6	-	-	-
SR02874	2.50	*Ss *cDNA	4.00E-05	TBN95TM-SSFH	-	-	-
SR00073	2.46	*Ce *hypo prot: confirmed	2.00E-26	Y43F8B.1a	-	-	-
SR00604	2.46	*Ce *PAB-1	4.00E-15	Y106G6H.2a	regulation of protein activity, epigenetic	-	-
SR01164	2.43	*Ss *EST	2.00E-06	TBN95TM-SSFH	-	-	-
SR02266	2.39	*Ce *hypo prot: partially confirmed	2.00E-23	R02F2.8	-	-	-
SR00717	2.39	*Ce *ATP synthase complex	4.00E-72	R53.4	-	-	-
SR01779	2.36	*Ce *hypo prot: predicted	7.00E-17	M60.7	-	-	-
SR02390	2.36	-	-	-	-	-	-
SR00889	2.34	*Ce *ASP-3	8.00E-76	H22K11.1	induction of non-apoptotic programmed cell death, proteolysis and peptidolysis	aspartic-type endopeptidase activity	lysosome
SR00607	2.34	*Ce *ASB-2	2.00E-99	F02E8.1	embryogenesis and morphogenesis	-	-
SR00704	2.29	*Ce *hypo prot: confirmed	5.00E-54	E04A4.7	-	-	-
SR01197	2.29	*Ce *pancreatic trypsin inhibitor	3.00E-11	W01F3.3	-	-	-
SR00813	2.29	*Ce *ZYX-1	2.00E-65	F42G4.3a	cell adhesion, signal transduction, cell-cell signalling	-	integral to plasma membrane
SR01881	2.28	*Ce *hypo prot: partially confirmed	9.00E-52	Y40B10A.2	-	-	-
SR00998	2.27	*Ce *MLC-2	3.00E-91	C36E6.5	regulation of striated muscle contraction, regulation of muscle contraction	structural constituent of muscle	cytoskeleton
SR00241	2.27	*Ce *LEV-11	4.00E-114	Y105E8B.1a	regulation of muscle contraction	unknown	cytoskeleton, cytoskeleton
SR00192	2.25	*Ce *neuroendocrine protein 7B2	1.00E-38	T03D8.3	-	GTP binding, enzyme activator activity	secretory vesicle
SR02882	2.25	*Ce *ALG-1	1.00E-93	F48F7.1	embryonic development, larval development	unknown	-
SR01335	2.22	*Ce *PAS-5	4.00E-18	F25H2.9	-	-	26S proteasome
SR00164	2.21	*Ce *hypo prot: confirmed	3.00E-71	Y37D8A.14	-	cytochrome-c oxidase activity, electron transporter activity	-
SR00231	2.18	-	-	-	-	-	-
SR00903	2.18	*Ss *EST	9.00E-78	TBN95TM-SSFH	-	-	-
SR01737	2.17	*Ss *EST	6.00E-09	TBN95TM-SSFH	-	-	-
SR02037	2.17	*Ce *IFB-1	8.00E-42	F10C1.2a	-	-	lamin filament
SR02982	2.17	*Ce *hypo prot: partially confirmed	4.00E-38	Y105E8A.19	tyrosyl-tRNA aminoacylation, apoptosis, cell motility, protein biosynthesis	tyrosine-tRNA ligase activity, signal transducer activity, interleukin-8 receptor binding	soluble fraction
SR02952	2.16	*Ce *hypo prot: partially confirmed	3.00E-41	K07E1.1	-	-	-
SR00038	2.13	*Ce *UBQ-1	7.00E-26	F25B5.4a	proteolysis and peptidolysis, protein metabolism	-	ribosome
SR02710	2.11	*Ce *hypo prot: predicted	7.00E-38	Y59A8B.23	protein amino acid phosphorylation, response to stress	receptor signalling protein serine/threonine kinase activity, ATP binding	-
SR00308	2.09	*Ce *EAT-6	4.00E-18	B0365.3	-	sodium/potassium-exchanging ATPase activity	membrane fraction
SR00918	2.09	*Ce *FTT-2	4.00E-13	F52D10.3a	chromosome segregation, learning and/or memory, olfactory learning	protein kinase C inhibitor activity	-
SR00906	2.06	*Ce *hypo prot: partially confirmed	7.00E-84	Y53G8AR.3	-	-	-

^1^The mean cluster iL3 : L1 difference in expression.

^2^The most significant BLAST alignment for each cluster, the BLAST score, E, and GenBank accession number.

^3^Gene ontology slim term, [52].

^4^- = no alignment or assignment, as appropriate.

^5^*Ce, C. elegans; Mm, Mus musculus; Ov, Onchocerca volvulus; Pt, Parastrongyloides trichosuri; Ss, Strongyloides stercoralis.*

^6^hypo prot – hypothetical protein; predicted – ORF predicted by GeneFinder in WormBase [53]; partially confirmed – partial sequence cloned; confirmed – full-length sequence cloned.

^7^NK, not known.

**Table 4:** The 37 L1-up clusters (Table 1) showing the mean cluster fold change between L1 and iL3 stages, the most significant BLAST alignment and the GO slim terms, arranged by descending L1 : iL3 difference in expression.

**Cluster**	**Expression**^**1**^	**BLAST alignment**^**2**^	**E**^**2**^	**Accession number**^**2**^	**Gene ontology (GO) slim term**^**3**^		
					
					**Process**	**Function**	**Component**
SR01196	3.65	*Ce*^4 ^hypo prot^5^: confirmed	2.00E-16	Y34B4A.6	-^6^	-	-
SR00880	3.13	*Ce *RPS-29	4.00E-28	B0412.4	embryogenesis & morphogenesis	-	ribosome
SR01112	2.87	-^6^	-	-	-	-	-
SR00783	2.78	*Ce *RPS-21	2.00E-33	F37C12.11	protein biosynthesis	constituent of ribosome	ribosome
SR00048	2.77	*Ce *RPL-30	2.00E-39	Y106G6H.3	-	-	-
SR02498	2.77	-	-	-	-	-	-
SR00689	2.64	*Ce *RPL-38	7.00E-17	C06B8.8	protein biosynthesis	RNA binding, constituent of ribosome	ribosome
SR02484	2.57	-	-	-	-	-	-
SR01003	2.54	*Ce *RPS-14	1.00E-79	F37C12.9	protein biosynthesis & metabolism	-	ribosome
SR01012	2.50	*Ce *RPS-15	8.00E-67	F36A2.6	embryogenesis & morphogenesis	-	-
SR00216	2.43	*Ce *hypo prot: confirmed	2.00E-33	C14A4.1	embryogenesis & morphogenesis	-	phycobilisome
SR00059	2.38	*Ce *RPS-10	4.00E-42	D1007.6	embryogenesis & morphogenesis	-	-
SR00938	2.38	*Ce *RPL-33	2.00E-37	F10E7.7	-	tRNA binding	-
SR00178	2.35	*Ce *hypo prot: predicted	7.00E-12	VK10D6R.1	-	-	-
SR02516	2.35	*Ce *VHA-2	3.00E-16	P34546	proton transport	-	integral to membrane
SR00926	2.31	*Ce *RPL-23	2.00E-76	B0336.10	protein biosynthesis & metabolism	-	ribosome
SR00943	2.31	*Ce *RPL-14	4.00E-44	C04F12.4	embryogenesis & morphogenesis	-	ribosome
SR00211	2.30	*Ce *RPL-31	4.00E-45	W09C5.6a	protein biosynthesis	constituent of ribosome	ribosome
SR00804	2.30	*Ce *RPS-11	5.00E-62	F40F11.1	embryogenesis & morphogenesis	-	ribosome
SR00871	2.30	*Ce *LYS-4	6.00E-60	F58B3.1	-	-	-
SR00951	2.28	*Ce *RPL-37	1.00E-39	W01D2.1	-	-	-
SR01225	2.28	*Ce *hypo prot: confirmed	3.00E-41	C30C11.1	protein biosynthesis	-	mitochondrial ribosome
SR00012	2.27	*Ce *COL-34	1.00E-15	F36A4.10	-	-	-
SR02773	2.27	*Ce *adenylate kinase	1.00E-92	P34346	-	adenylate kinase activity	-
SR01008	2.23	*Ce *RPS-26	9.00E-61	F39B2.6	larval development	-	-
SR01007	2.22	*Ce *RPS-4	1.00E-111	Y43B11AR.4	protein biosynthesis	constituent of ribosome	ribosome
SR00978	2.20	*Ce *RPS-13	2.00E-80	C16A3.9	protein biosynthesis	constituent of ribosome	ribosome
SR01030	2.20	*Ce *RPS-5	3.00E-108	T05E11.1	protein biosynthesis	constituent of ribosome	ribosome
SR00801	2.18	*Ce *hypo prot: confirmed	9.00E-53	Y82E9BR.3	-	-	integral to membrane
SR00915	2.08	*Ce *RPL-29	1.00E-18	B0513.3	protein biosynthesis	RNA binding, constituent of ribosome	ribosome
SR01068	2.07	*Ce *RPL-1	4.00E-110	Y71F9AL.13a	protein biosynthesis	constituent of ribosome	ribosome
SR02436	2.07	*Ce *hypo prot: confirmed	1.00E-57	Q9U330	dephosphorylation, phosphoinositide metabolism, dephosphorylation	inositol/phosphatidylinositol phosphatase activity	integral to golgi and endoplasmic reticulum membrane
SR00026	2.06	*Ce*^4 ^RPA-1	2.00E-45	Y37E3.7	-	-	-
SR00886	2.06	*Ce *RPL-34	1.00E-32	C42C1.14	protein biosynthesis	constituent of ribosome	ribosome
SR00811	2.04	*Ce *RPS-20	9.00E-52	Y105E8A.16	protein biosynthesis & metabolism	-	ribosome
SR00588	2.04	*Ce *COL-156	2.00E-76	F57B7.3	cuticle biosynthesis	constituent of cuticle	extracellular
SR02281	2.00	*Ce *hypo prot: confirmed	3.00E-40	Q21732	-	-	-

^1^The mean cluster L1 : iL3 difference in expression.

^2 ^The most significant BLAST alignment for each cluster, the BLAST score, E, and GenBank accession number.

^3^Gene ontology slim term, [52].

^4 ^*Ce: C. elegans*

^5^hypo prot – hypothetical protein; predicted – ORF predicted by GeneFinder in WormBase [53]; partially confirmed – partial sequence cloned; confirmed – full-length sequence cloned.

^6^- = no assignment or alignment, as appropriate.

**Table 5:** The number of ESTs, contigs and clusters differently expressed in L2 direct and L2 indirect stages and those with a two-fold or greater difference in expression.

	**L2 direct-up**	**L2 indirect-up**	**Total**
**EST**^**1**^	246	429	675
**≥ 2-fold**^**2**^	1	4	5
**Contig**^**3**^	105	320	425
**≥ 2-fold**^**4**^	1	2	3
**Cluster**^**3**^	93	311	404
**≥ 2-fold**^**4**^	1	2	3

^1^These ESTs are represented by 107 and 95 (L2 direct-up) and 323 and 316 (L2 indirect-up) contigs and clusters respectively.

^2^Number of ESTs that have a ratio of expression that is ≥ 2; these ESTs are represented by 1 (L2 direct-up) and 3 (L2 indirect-up) contigs and clusters respectively.

^3^Number of contigs or clusters, respectively, in which at least one EST member (for which there were data above the quality threshold) had a significantly different ratio of expression.

^4^Number of contigs or clusters, respectively, in which at least one EST member (for which there were data above the quality threshold) has a significantly different ratio of expression that is ≥ 2.

**Table 6:** A summary of the three L2 clusters whose expression is significantly different between indirectly and directly developing L2s.

**Cluster**	**Expression**^**1**^	**BLAST alignment**^**2**^	**E**^**2**^	**Accession number**^**2**^
SR02363	2.08	*Ce *hypo prot: partially confirmed^3^	8.00E-17	Y39B6A.1
SR00170	2.58	*Sm *ORF-3^4^	3.00E-05	AAA29909.1
SR00374	2.02	*Ce *probable peroxiredoxin	1.00E-81	R07E5.2

^1^The mean cluster difference in expression. L2 direct-up: SR02363. L2 indirect-up: SR00170, SR00374.

^2^The most significant BLAST alignment for each cluster, the BLAST score, E, and GenBank accession number.

^3^*C. elegans *hypothetical protein predicted by GeneFinder in WormBase [53]; partial sequence cloned.

^4^Conceptual translation of an *Schistosoma mansoni *sequence [54].

**Table 7:** Reverse transcriptase-PCR analysis of the expression of clusters and contigs differentially expressed between the directly and indirectly developing L2s.

**Cluster**^**1**^	**Fold**^**1**^	**Contig**^**1**^	**Fold**^**1**^	**RT-PCR Mean (SD)**^**2**^
SR02363	2.08	SR02363	2.08	1.45 (0.46)
SR00009	1.69	SR00027	1.69	0.76 (0.45)
SR00113	1.60	SR00113	1.58	1.30 (0.67)
SR00113	1.60	SR00987	1.63	1.21 (0.18)
SR00013	1.25	SR00053	1.26	0.95 (0.48)
SR02856	1.48	SR02856	1.48	2.69 (2.06)
SR00395	1.46	SR00395	1.46	1.81 (2)
SR00012	1.40	SR00989	1.45	0.66 (0.17)
SR00012	1.40	SR00665	1.39	0.99 (0.59)
SR01030	1.39	SR01030	1.39	1.09 (0.6)
SR00008	1.31	SR00752	1.31	1.81 (1.01)
SR01870	1.34	SR01870	1.34	1.59 (2.06)
SR00591	1.30	SR00591	1.30	0.75 (0.33)
SR00012	1.40	SR00955	1.28	1.30 (0.35)
SR00055	1.23	SR00962	1.23	0.97 (0.14)
SR00112	1.21	SR00112	1.21	3.86 (5.72)
SR00950	1.18	SR00950	1.18	1.46 (1.45)
SR01054	1.18	SR01054	1.18	2.98 (3.91)
SR00611	1.16	SR00611	1.16	4.38 (2.89)
SR00038	1.22	SR00038	1.13	0.97 (0.51)
SR00205	1.01	SR00205	1.01	0.38 (0.33)
**SR00170**	**2.58**	**SR00170**	**2.58**	**6.18 (1.76)**
**SR00374**	**2.02**	**SR00374**	**2.02**	**7.27 (4.57)**
**SR00351**	**1.73**	**SR00931**	**1.83**	**12.9 (20.3)**
**SR00713**	**1.74**	**SR00713**	**1.74**	**1.13 (0.36)**
**SR00775**	**1.68**	**SR00775**	**1.68**	**6.28 (8.64)**
**SR00199**	**1.45**	**SR00199**	**1.45**	**1.71 (0.22)**
**SR01250**	**1.38**	**SR01250**	**1.38**	**3.51 (0)**
**SR00799**	**1.35**	**SR00799**	**1.35**	**4.38 (4.28)**
**SR00811**	**1.28**	**SR00811**	**1.28**	**0.91 (0.3)**
**SR00092**	**1.21**	**SR00092**	**1.21**	**0.99 (0.24)**
**SR02792**	**1.21**	**SR02792**	**1.21**	**6.56 (7.21)**
**SR00721**	**1.19**	**SR00721**	**1.19**	**1.06 (0.27)**
**SR02972**	**1.17**	**SR02972**	**1.17**	**14.8 (23.1)**
**SR00994**	**1.16**	**SR00994**	**1.16**	**1.16 (0.43)**
**SR01156**	**1.57**	**SR01156**	**1.57**	**2.04 (2.06)**
**SR02019**	**1.84**	**SR02019**	**1.84**	**2.85 (3.94)**
**SR00178**	**1.39**	**SR00178**	**1.39**	**1.09 (0.11)**
**SR00342**	**1.14**	**SR00543**	**1.14**	**10.8 (1.5)**
**SR02917**	**1.1**	**SR02917**	**1.1**	**26.5 (41.6)**
**SR00615**	**1.15**	**SR00615**	**1.1**	**1.76 (0.65)**
**SR02394**	**1.07**	**SR02394**	**1.07**	**8.72 (1.75)**
**SR00817**	**1.06**	**SR00817**	**1.06**	**13.4 (1.11)**
**SR00266**	**1.06**	**SR00266**	**1.06**	**2.55 (2.5)**
**SR00451**	**1.02**	**SR00451**	**1.02**	**11.1 (16.2)**
SR00113*	1.6	SR00113	1.58	1.23 (0.87)
SR00015*	1.1	SR00015	1.1	0.25 (0.01)

^1^Mean cluster and contig fold difference in expression between indirectly and directly developing L2 stages measured by microarrays for L2 direct-up (normal text) and L2 indirect-up (bold text) clusters and contigs and for reference genes (*).

^2 ^The mean (SD) fold difference in expression as measured by RT-PCR is calculated as direct/indirect for the L2 direct-up (normal text) contigs and clusters and for the reference genes (*) and indirect/direct for the L2 indirect-up (bold text) contigs and clusters.

**Table 8:** The expression of *S. ratti *genes with and without significant alignment to *C. elegans *genes with RNAi-knockdown phenotypes.

***C. elegans *RNAi phenotype**	**BLAST alignment**^**1**^	**Expression**^**2**^	**p**^**3**^
**Growth**	481 alignments	L1-up (1.18)	0.0001
	1,422 no alignment	iL3-up (1.12)	
**Larval arrest**	342 alignments	L1-up (1.26)	0.0001
	1,561 no alignment	iL3-up (1.10)	
**Morphology**	272 alignments	L1-up (1.22)	0.001
	1,631 no alignment	iL3-up (1.09)	
**Movement**	187 alignments	L1-up (1.17)	0.01
	1,716 no alignment	iL3-up (1.06)	
**Reproduction**	340 alignments	L1-up (1.16)	0.001
	1,563 no alignment	iL3-up (1.09)	
**Total**^**4**^	1,622 alignments	L1-up (1.18)	0.00001
	281 no alignment	iL3-up (1.16)	

^1^Number of *S. ratti *ESTs with or without significant BLAST alignment to *C. elegans *genes with RNAi phenotypes.

^2^The direction of the difference in expression (*i.e. *L1- or iL3-up) and, shown in parentheses, the mean ratio of expression.

^3^The significance of the difference of the mean levels of expression for the ESTs with and without significant BLAST alignments.

^4^All RNAi phenotypes combined. Note, that 511 *S. ratti *ESTs had significant alignment to a *C. elegans *gene that had an RNAi phenotype in more than one phenotypic class.

**Table 9:** The expression of *S. ratti *contigs with and without significant alignment to *S. stercoralis *L1- or iL3- specific or – biased contigs.

***S. stercoralis *contig expression**^**1**^	**BLAST alignments**^**2**^	**Expression**^**3**^	**p**^**4**^
**L1-biased**	114 alignments	iL3-up (1.23)	0.001
	874 no alignment	L1-up (1.09)	
**iL3-biased**	133 alignments	iL3-up (1.21)	0.01
	855 no alignment	iL3-up (1.03)	
**L1-specific**	349 alignments	iL3-up (1.22)	0.05
	639 no alignment	iL3-up (1.13)	
**iL3-specific**	254 alignments	iL3-up (1.21)	1.0
	734 no alignment	iL3-up (1.12)	

^1^Mitreva pers. comm. [17].

^2^Number of *S. ratti *contigs with or without a significant BLAST alignment to the *S. stercoralis *contigs in the respective categories.

^3^The direction of the difference in expression (*i.e. *L1- or iL3-up) and, shown in parentheses, the mean ratio of expression.

^4^The significance of the difference of the mean levels of expression for the ESTs with and without significant BLAST alignments.
