# Supplementary material for: A microarray analysis of gene expression in the free-living stages of the parasitic nematode Strongyloides ratti
Source: BMC Genomics. 2006 Jun 19;7:157. doi: 10.1186/1471-2164-7-157 (PMC1525192; doi:10.1186/1471-2164-7-157)
Supplement: Additional file 3 — A table detailing PCR primer sequences. [file 1471-2164-7-157-S3.doc]

# Additional data file 3

#### PCR primer sequences

| Primer | **5’ 3’** |
| --- | --- |
| Sp6 | atttaggtgacactata |
| T7 | taatacgactcactataggg |
| TopoFor | tcggatccactagtaacggc |
| TopoRev | ccgccagtgtgatggatatc |
